# Bi-FPNFAS: Bi-Directional Feature Pyramid Network for Pixel-Wise Face Anti-Spoofing by Leveraging Fourier Spectra

**DOI:** 10.3390/s21082799

**Published:** 2021-04-15

**Authors:** Koushik Roy, Md. Hasan, Labiba Rupty, Md. Sourave Hossain, Shirshajit Sengupta, Shehzad Noor Taus, Nabeel Mohammed

**Affiliations:** 1 Gaze Pte. Ltd., Singapore 068914, Singapore; koushik@gaze.ai (K.R.); hasan@gaze.ai (M.H.); labiba@gaze.ai (L.R.); sourave@gaze.ai (M.S.H.); shirsho@gaze.ai (S.S.); t@gaze.ai (S.N.T.); 2 Department of Electrical and Computer Engineering, North South University, Dhaka 1229, Bangladesh

**Keywords:** liveness sensing, counter-spoofing detection, biometric sensors, biometric authentication, face anti-spoof, Fourier spectra, neural networks, bidirectional feature pyramid networks

## Abstract

The emergence of biometric-based authentication using modern sensors on electronic devices has led to an escalated use of face recognition technologies. While these technologies may seem intriguing, they are accompanied by numerous implicit drawbacks. In this paper, we look into the problem of face anti-spoofing (FAS) on a frame level in an attempt to ameliorate the risks of face-spoofed attacks in biometric authentication processes. We employed a bi-directional feature pyramid network (BiFPN) that is used for convolutional multi-scaled feature extraction on the EfficientDet detection architecture, which is novel to the task of FAS. We further use these convolutional multi-scaled features in order to perform deep pixel-wise supervision. For all of our experiments, we performed evaluations across all major datasets and attained competitive results for the majority of the cases. Additionally, we showed that introducing an auxiliary self-supervision branch tasked with reconstructing the inputs in the frequency domain demonstrates an average classification error rate (ACER) of 2.92% on Protocol IV of the OULU-NPU dataset, which is significantly better than the currently available published works on pixel-wise face anti-spoofing. Moreover, following the procedures of prior works, we performed inter-dataset testing, which further consolidated the generalizability of the proposed models, as they showed optimum results across various sensors without any fine-tuning procedures.

## 1. Introduction

**Figure 1:**
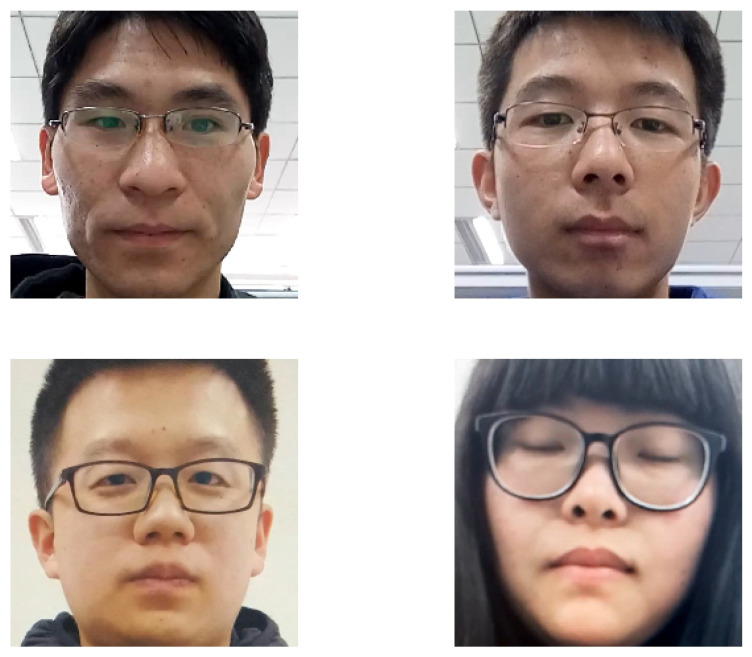
Samples of bonafide and spoofed images from the OULU-NPU [14] dataset, which illuminate the difficulty of visually discerning the two classes. The two images on the first row represent the bonafide samples, and the second row represents two spoofed samples.

The advent of popular face recognition technologies [1,2,3] in recent years has been accompanied by a greater scope of their applications. The dominant usage of these applications is based on biometric authentication, which is commonly found as the face-unlocking of smartphones or websites [4,5]. The extensive usage of this technology has exhibited vulnerabilities and proneness to various forms of attacks, such as adversarial face attacks, face manipulation attacks, and face-spoofing attacks [6,7]. Face-spoofing attacks are a physical modality of presentation attacks (PAs), which include paper, video replay, 3D mask, and makeup attacks. To elaborate, mere printouts of faces or video clips of faces performing various actions would be sufficient to fool a face recognition model, as shown in Figure 1. Hence, the need for face anti-spoofing technology has emerged in order to make face recognition models resistant to PAs.

Earlier approaches to face anti-spoofing (FAS) included the usage of hand-crafted features [8,9,10,11,12]; however, these models often failed to be generalized for images with the slightest variance in environmental settings (light, orientation, etc.). Another variant of FAS models requires users to continuously send feedbacks [13] to the system with specific cues, such as eye blinking, head movements, smiling, etc. However, this approach is flawed, as these cues can be easily reproduced with video replay attacks.

Recent approaches to FAS have made use of deep features that are often extracted from a convolutional neural network (CNN) [15] in an attempt to overcome the earlier problems. Furthermore, the usage of pixel-wise supervision over the complete convolutional feature on the euclidean space, as well as the angular space [16,17], has shown competitive results as well. The solution proposed by Yu et al. [18] built upon the idea of pixel-wise supervision on multiple scales of a Resnet [19] backbone. However, though this idea used a multi-scaled form of supervision, it did not leverage a feature pyramid network (FPN) [20], which can be used as a feature extractor to produce a number of multi-scaled feature maps from an input image, thus adding further contextual information to the prediction model and making it semantically robust.

In this paper, we aim to fill this gap by using a multi-scaled feature extractor, the bi-directional feature pyramid network (BiFPN)—primarily for the FAS problem—in an effort to extract multi-scaled features while also coupled with the EfficientNet [21] feature extractor. While prior works have shown the significance of texture-based features [22] for FAS, we hypothesize that due to the working principle of BiFPN, we could potentially extract features that would contain textural information that is imperative for this task. We assume that the introduction of BiFPN embodies specific cues for spoofed features resulting from receiving accurate responses when using similar samples of different sizes. This would confirm the subsistence of our initial assumption. In addition to the prior motivation of leveraging texture-based features, following [23], we also find that using Fourier-based features is intuitive for FAS, as we observed a higher number of high-frequency components for bonafide samples, but the opposite for attack samples.

We propose two variants of our FAS pipeline. Firstly, we show a baseline architecture that performs pixel-wise supervision by leveraging the BiFPN. We further extend this idea by combining an auxiliary branch that performs self-supervision on the frequency domain. Using the ideas mentioned above, we performed evaluations on multiple benchmark datasets and achieved competitive results for several protocols compared with other pixel-wise classification papers [16,17]. To summarize, the contributions of this paper are as follows:

- We propose a multi-scaled approach to face anti-spoofing, Bi-FAS, which uses a bi-directional feature pyramid network.
- We find that among the five different pyramid features, the inclusion of two larger pyramids containing high-level information demonstrates negligible improvements.
- We extend our previous approach based on BiFPN by introducing a self-supervised branch optimized on the frequency domain using a reconstruction loss. We refer to this model as Bi-FAS-S throughout the rest of this paper.

The remainder of the paper is structured as follows. The following section outlines a brief literature review of the past and present approaches used to combat face anti-spoofing. Next, we give an overview of the datasets and the metrics in the Materials section. We then discuss our methodology and explain our proposed architectures in the Methods section. Next, we use the Experiments and Results section to describe and present all the aspects of our experiments. In the Discussion section, we show an in-depth analysis of the results of our experiments. Finally, we give our concluding remarks and describe our future ideas in the closing section.

## 2. Related Works

This section outlines all of the literature relevant to this paper. We describe multiple approaches to FAS, from those that leverage handcrafted features to the newer CNN-based state-of-the-art (SOTA) models based on various forms of supervision. Although prior literature shows that FAS relies heavily on handcrafted features, we also describe our multi-scaled feature extractors that we use in this paper, as well as the literature relevant to Fourier-based FAS.

### 2.1. Face Anti-Spoofing

FAS can be incorporated into two different categories. One requires a single frame, and the other uses multiple frames containing temporal information for performing the task. Classical approaches to face anti-spoofing include the usage of traditional algorithms, such as Local Binary Pattern (LBP) [8,24], Histogram of Oriented Gradients (HOG) [9,24,25], Difference of Gaussian (DoG) [26,27], and Gabor Wavelets [28]. These algorithms are used to extract various features, which are passed on to a feature learner, possibly a support vector machine (SVM) [29], for the classification task. While these algorithms tend to work under a frame-level condition, there are several approaches where visual cues, such as eye blinking [30,31] and dynamic texture [32], can be used to detect spoofing at a video level. However, the caveat for these features is that they are susceptible to a lack of generalization, as evidenced by their testing metrics, and they eventually make high volumes of data a necessity for this task. Still, research on FAS has come a long way, and the CNN-based approaches have turned out to be the current norm. The authors of the Central Difference Convolutional Network (CDCN) paper [33] proposed a novel approach for frame-level FAS based on central difference convolution (CDC). The CDC is claimed to be sensitive to intricate patterns through depth, gradients, and intensity. The authors also showed an improved version of their proposed model by performing the Neural Architecture Search operation. The CDCN model has outperformed all of the mentioned approaches and holds the current SOTA scores on all major benchmark datasets. Yu et al. [18] improved upon the idea of pixel-wise supervision and proposed a novel pyramid-like model in the form of the extraction of multi-scaled features from a deep backbone. They further coupled this idea with the depth-based features from the CDCN paper to propose a second approach. They reported a competitive mean average classification error rate (ACER) of 4.8 on Protocol IV of the OULU-NPU dataset. In the following sub-section, we discuss CNN-based approaches that utilize a form of pixel-wise supervision for FAS.

### 2.2. Pixel-Wise Supervision for FAS

In the realm of FAS, the term pixel-wise supervision can be referred to as a model focusing on a synthesized feature map that is a bi-product of a feature extractor [16]. This method has led the FAS model in order to learn shared representations of various patch-level cues that are significant for this task [16]. DeepPixBis [16] proposes an FAS framework that aims to mitigate the need for temporal information by using a DenseNet [34] backbone to extract deep features embedded in a 14 × 14 convolution map. This feature map is later used to perform a pixel-wise loss calculation. The flattened 14 × 14 map is further fed to a fully connected layer sequenced with a sigmoid layer, which outputs a probability for the spoof class. The A-DeepPixBis [17] paper was built upon the DeepPixBis idea, which supervised based on two branches (one pixel-wise branch); however, they performed the computations on the angular space by proposing a new angular binary cross-entropy loss function, as shown in Equation (1).

(1) $$L_{\text{AM-BCE}}=-\frac{1}{N}\sum_{i=1}^{N}p_{i}log\left(\sigma \left(\cos\left(\theta_{i}+m\right)\right)\right)+\left(1-p_{i}\right)log\left(1-\sigma (\cos\theta_{i})\right)$$

In Equation (1), $p_{i}$ refers to the ground truth, and θ, for a sample *i*, is a feature map after applying convolution on the angular space over the 14 × 14 feature extracted from the DenseNet [34] feature extractor. The term *m* is an added margin to enforce the separation of decision boundaries in the angular space. The A-DeepPixBis paper achieved competitive scores on the hardest protocol of the OULU-NPU dataset [14].

### 2.3. Fourier-Spectra-Based FAS

Li et al. [23] proposed a high-frequency descriptor (HFD) that leveraged the idea of Fourier transformation on a face. It was based on the hypothesis that the median of HFDs for a sequence of images, if lower than a specific threshold, should be classified as a spoofed sample. If not, it used the standard deviation of the energy values (frequency dynamic descriptor) that were predefined over the sequence of images. The frequency dynamic descriptor quantity was used to finally classify the image. We took inspiration from this paper for our hypothesis with the use of a self-supervised branch based on the Fourier spectra.

### 2.4. Multi-Scaled Feature Representation

The representation of an image projected into features of multiple scales has been a trend in recent CNN-based object detectors [35,36]. These features are generally extracted from a deep backbone network, which outputs the features from each of their consecutive layers in a pyramid-like approach. The feature pyramid network [20] was proposed as a top-down multi-scale feature extractor for extracting semantically rich features, which are used in object detectors, such as Faster R-CNN [35]. This solves the fundamental problem of recognizing images on multiple scales, thus enabling a detector to predict minuscule objects as well as objects of significant size. The PANet [37] added a bottom-up information flow to the original top-down pyramid approach of the FPN. NAS-FPN [38] is one of the recently proposed feature pyramid networks. Although it is very effective, this model comes with operations such as Neural Architecture Search, which requires very high computational power and results in inconsistent architectures.

### 2.5. EfficientDet

EfficientDet [39] is a new family of efficient and scalable object detection modules that were built using EfficientNets [21]. Tan et al. [39] incorporated a novel feature extractor network, BiFPN, and EfficientNet to achieve SOTA performance on object detection tasks while being up to 9 times smaller than current SOTA models.

A typical object detection pipeline consists of three parts: a backbone network that is responsible for extracting features from the input image, an FPN [20] that takes features from different layers of the backbone network, and a classification/box network for the final output. EfficientDet [39] uses a BiFPN to fuse features coming from a different level of the backbone network and a variant of EfficientNet as the backbone.

In their paper, the authors of [39] showed that previously used backbone networks, such as ResNets [19], ResNexts [40], DenseNets [34], or MobileNets [41], are generally not powerful enough or not efficient enough. For instance, they compared EfficientNet-B3 with ResNet-50 and showed that it is more accurate and almost 20% more efficient than ResNet-50. They also showed one flaw of FPN: that it works in a top-down fashion, and is therefore limited by one-way information flow. Although there is an alternative in the form of PANet [37], which considers both top-down and bottom-up feature fusion, it adds more cost to the network.

In order to address this issue, the authors of [39] proposed a novel FPN network called BiFPN, which fuses multi-level features from the backbone in both a top-down and bottom-up manner. To further reduce the computation, the authors of [39] used separable convolutions instead of plain convolutions. With these optimizations in place, the EfficientDet model further improved the accuracy by 4% while increasing the efficiency by up to 50%. For the two architectures proposed in this paper, we utilized the aforementioned BiFPN module as part of the multi-scaled feature extractor of the input sample.

Our work in this paper heavily leverages the idea of using deep pixel-wise features from the DeepPixBis and A-DeepPixBis papers, for which we use the EfficientNet model as the feature extractor and BiFPN for multi-scaled features; furthermore, we take influence from the features based on Fourier spectra—as mentioned earlier—to design a self-supervised auxiliary branch. We discuss the techniques elaborately in the Methodology section.

## 3. Materials

This section elaborates on the materials we used to perform all of the experiments. We begin with the descriptions of the OULU-NPU [14] and the Replay-Mobile [6] datasets. We also provide details about the metrics used in the evaluation processes.

### 3.1. Datasets

For all of the experiments conducted in this paper, we used two popular benchmark datasets for FAS and provide a brief description of them below.

OULU-NPU: This dataset [14] consists of 55 subjects; the videos were recorded with six different phone devices in three distinct environments in an attempt to replicate a real-world scenario. The attack samples are comprised of display attacks and print attacks, each with two variants. The total of 1980 bonafide videos and 3960 attack videos make this one of the most diverse and challenging datasets for this task. For better evaluation of the generalization of the FAS model, the creators of this dataset provided us with four different protocols, each serving a specific criterion. An overview of all the protocol configurations can be found in Table 1, and a description of the four protocols is as follows:

1. Protocol I evaluates the model’s invariance to different environments; the environments of the training and validation sets are different from the ones in the testing set.
2. Protocol II tests if the model is robust to various devices, with dissimilar devices in the training and the testing partitions.
3. Protocol III uses tests that consist of phones with various camera resolutions that are different from the resolutions present in the training and testing sets.
4. Protocol IV is a composition of all preceding constraints, but also with a smaller training set. This is undoubtedly the most challenging protocol [16] among the four.

Replay-Mobile: The Replay-Mobile dataset [6] consists of 1200 videos with 40 subjects. Two different illumination conditions are used in this dataset, ranging from well-lit to dimmed samples. Each subject was recorded in five background conditions with two different recording devices, an iPad Mini 2 and an LG-G4 phone. The attack samples are of two types—mattescreen, where a printed sample is presented on a high-resolution phone, and print attacks, where the digital photos are presented on an A4-sized paper. We used the grandtest protocol of the dataset to perform the global performance evaluation. This protocol uses 1040 videos with a train, dev, and test split with a 3:4:3 ratio.

### 3.2. Metrics

For the evaluation of our models, we used the ISO/IEC 30107-3 [42] certified metrics, which are the current standard for FAS and are used by popular FAS papers [16,33]. We used the attack presentation classification error rate (APCER) to measure the performance of the models on presentation attack instances (PAIs) and used the bonafide presentation classification error rate (BPCER) to measure the performance of the model on the bonafide images. We further calculated the average classification error rate (ACER), which is the mean of the APCER and BPCER. Moreover, for the experiments in this paper, APCER refers to the false-negative rate, where the negative class denotes an attack sample, as shown in Equation (2), where FN is the number of misclassified attack samples and TP is the number of correctly classified bonafide samples. The BPCER refers to the false-positive rate, where the positive class denotes a bonafide sample, as shown in Equation (3), where FP is the number of misclassified bonafide samples and TN is the number of correctly classified attack samples. The mathematical definition of the ACER is shown in Equation (4). We also used the generalized accuracy metric to prevent the model from overfitting.

(2) $$APCER=\frac{FN}{FN+TP}$$

(3) $$BPCER=\frac{FP}{FP+TN}$$

(4) $$ACER=\frac{APCER+BPCER}{2}$$

The inter-dataset results are reported by using the half-total error rate (HTER), where the HTER is the average of the false rejection rate (FRR) and the false acceptance rate (FAR), as shown in Equation (5). We also used the equal error rate (ERR) as per the implementation described by [43] to evaluate the Replay-Mobile dataset; as described by [43], in theory, the EER is defined as the point of intersection between the FAR and FRR. However, in practice, while performing experiments, it may not always be possible to find the “perfect” point of intersection due to numerical inconsistencies. Thus, we computed the absolute difference between the FRR and FAR to find the index, *m*, that denotes the closest pair of points between FAR and FRR (for multiple thresholds), and we further calculated the mean of the FRR and FAR at index *m* to find the EER. The calculation process is shown in Equation (6).

(5) $$HTER=\frac{FRR+FAR}{2}$$

(6) $$m=\underset{i}{\mathrm{argmin}}(|FRR_{i}-FAR_{i}\left|\right);\;\;EER=\frac{FRR_{m}+FAR_{m}}{2}$$

## 4. Methods

In this section, we discuss our proposed approach for the FAS task. Firstly, we discuss the overall pipeline, which elaborates on the preprocessing steps used to prepare the input samples for the FAS detection pipeline. Next, we elaborate on the two variants of our BiFPN model, which is designed for the classification of a spoofed or bonafide image.

### 4.1. Pipeline

**Figure 2:**
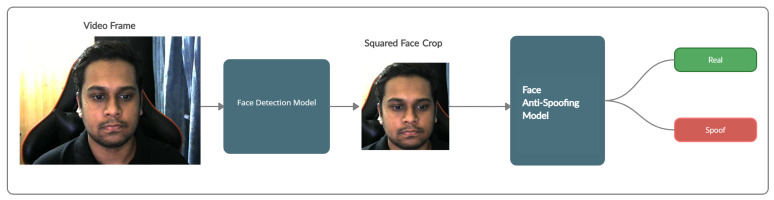
The overview of the proposed framework.

Our FAS pipeline, as shown in Figure 2, shows a high-level visualization of the overall process. From the figure, we can observe that our FAS pipeline is a composition of a face detection model that is used to extract the face crop of the video frame, which is a standard pre-processing step [14,16] for any architecture performing a downstream task relevant to facial information. Additionally, this process restricts the model from learning any background artifacts that may exist in the image. Therefore, for extraction, we use the RetinaFace [44] detection framework for the face crop extraction task. The red–green–blue (RGB) face crops are further resized to a resolution of 512 × 512, as we use a pre-trained feature extractor, EfficientNet [21], trained on this resolution over the ImageNet [45] dataset. This image is then passed on to our FAS model, which gives a probability score of the input being a real image. However, due to observations and extended experimentation, we found that rather than extracting a tight bounding box, if we selected a squared bounding box, our models showed noteworthy improvements during testing.

**Figure 3:**
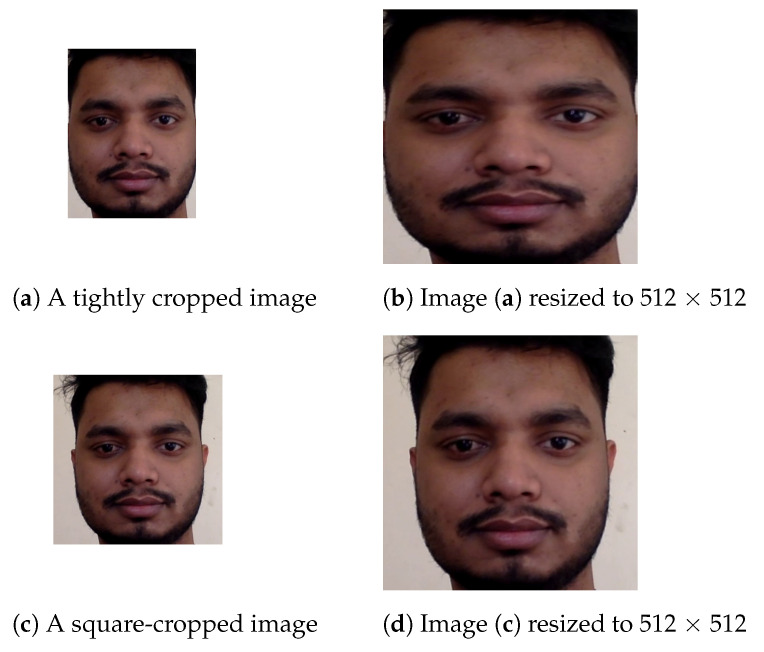
Representations of images using the two methods of cropping and their respective transformed image after resizing for model input. Image (**a**) demonstrates a tightly cropped image using the bounding boxes of RetinaFace [44], and (**b**) shows a sample where the bounding boxes of (**a**) were expanded to make a squared shape.

To further elaborate, any cropping that we perform on the full-frame of an image needs to be reshaped to 512 × 512, as per our model specifications. Hence, we used two forms of cropped faces for our experiments. Initially, we used the face crop bounding boxes returned by RetinaFace [44], as shown on Figure 3a, and further transformed them to 512 × 512, as shown in Figure 3b, for the model. However, we found that this transformation of resizing the image tended to be unnatural, as it modified the aspect ratio of the tightly cropped image and possibly aggravated textural features by introducing new artifacts in the image. On the other hand, if we used a squared bounding box of the face from the face detector, as shown in Figure 3c, and transformed it into 512 × 512, as shown in Figure 3d, we would not encounter any major changes in the aspect ratio of the image, thus potentially preserving any significant features of the image.

### 4.2. Feature Extractor—EfficientNet

EfficientNet [21] proposes a family of models that are efficient and accurate. While conventional architectures choose arbitrary scale factors for width, depth, and resolution, it proposes a compound coefficient to scale all three factors in a structured manner. With their uniform scaling method for each dimension, EfficientNet outperforms the SOTA models while maintaining up to 10 × efficiency for ImageNet [45].

In their study, they found that though scaling different dimensions of a model did improve performance with respect to the baseline counterpart (e.g., ResNet-18 and ResNet-100 [19]), scaling all of the dimensions in a balanced manner against available resources would provide the best overall performance. The EfficientNet model performed a grid search to determine the relationship between different scaling factors for all dimensions of the baseline network and the enforced resource constraint (e.g., 3 × more floating point operations per second). After that, they scaled the baseline network with the determined coefficient to get the targeted model.

The EfficientNet paper [21] shows that this scaling factor can be transferred to other network architectures as well. In their study, they observed a 1.4% ImageNet [45] accuracy improvement for the MobileNet model [41] and a 0.7% ImageNet accuracy improvement for the ResNet model [19]. The compound scaling method uses a compound coefficient ϕ, which uniformly scales the network’s width, depth, and resolution in a structured way. Following Equation (7), we show how this coefficient is used to scale all the dimensions.

(7) $$\begin{array}{cc} depth:d & =\alpha^{\phi }\\ width:w & =\beta^{\phi }\\ resolution:r & =\gamma^{\phi } \end{array}$$

The α,β,γ in Equation (7) are constants that can be determined by a grid search. In addition, α≥1, β≥1, and γ≥1.

### 4.3. Baseline Model

**Figure 4:**
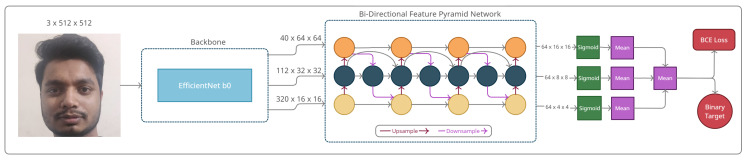
The architecture of our baseline bi-directional feature pyramid network (BiFPN) model, Bi-FAS.

In this paper, we propose two disparate approaches to FAS, but with similar assumptions. We hypothesized that leveraging weighted multi-scaled features and the aggregation of those features at different resolutions contribute to the intricate information required for this task. Due to the consistent results using similar images with different resolutions, we hypothesized that the features of the BiFPN contain texture-based cues, which may be essential for FAS. We first discuss our baseline BiFPN model (Bi-FAS), which is presented in Figure 4. We used the EfficientNet [21] architecture as our backbone feature extractor, particularly the b0 variant, which was the smallest model in terms of the number of trainable parameters. We mainly employed this backbone to extract features that would be uniformly scaled to multiple depths, widths, or resolutions for better fine-grained patterns. As depicted in Figure 4, we passed an RGB image to our EfficientNet feature extractor, which computed the features in multiple levels through convolutional layers. Outputs from the different levels of the backbone were used as an input to the BiFPN for the feature fusion process [39]. From the backbone, we used outputs from levels 3, 4, and 5 consisting of the shapes (40 × 64 × 64), (112 × 32 × 32), and (20 × 16 × 16), respectively. Throughout all of our experiments, we initialized the backbone with the pre-trained ImageNet [45] weights to restrict the model from making random predictions during the initial training phase. Additionally, during testing, our experiments showed that initializing the models with random weights led to inferior performance. We utilized the outputs of the feature extractor to feed it to the BiFPN, a weighted multi-scaled feature extractor, as shown in Equation (8).

(8) $$P_{n+1}=P_{n}+\Theta \left(P_{n}\right):\forall n\;\left(n\in \Upsilon \right)$$

The BiFPN outputs the features on five different scales ranging from $P_{1}$ to $P_{5}$, as presented in Table 2, where Θ refers to the convolutional layer of the *n*th pyramid and set Υ denotes the indexes of the pyramids used in the BiFPN model. However, our initial experiments demonstrated no utility of including the pyramids $P_{1}$ and $P_{2}$, which are two high-level feature pyramids. Thus, we left out pyramids $P_{1}$ and $P_{2}$ for all further experiments conducted in this paper. We computed the pixel-wise probabilities by applying the sigmoid operator, computed the mean probability score from all three pyramids using Equation (9), and obtained $p_{i}\;\epsilon \;R$. We used the three probability scores, $p_{i}$, to calculate the final probability score, *z*, in Equation (10), similarly to the first branch in the DeepPix and A-DeepPix papers [16,17].

(9) $$p_{i}=\frac{1}{T}\sum_{w=1}^{64}\sum_{x=1}^{T}\sum_{y=1}^{T}\frac{1}{1+e^{-P_{w,x,y}}}:\forall i\;\left(i\in \left\{3,4,5\right\}\right)\;;T=\left\{16,8,4\right\}$$

(10) $$z=\frac{1}{3}\sum_{i=3}^{5}p_{i}$$

This probability score determines the “realness” classification of this task. Subsequently, we optimized the model based on the probability scores using the binary cross-entropy loss function during the training phase.

(11) $$l=-\frac{1}{N}\sum_{i=1}^{N}t_{i}\;\cdot \;log\left(s_{i}\right)+\left(1-t_{i}\right)\;\cdot \;log\left(1-s_{i}\right)$$

For the binary cross-entropy loss defined on Equation (11), *N* denotes the total number of samples in the batch, *t* refers to the ground truth, and *s* refers to the *z* value of the *i*th sample.

### 4.4. Self Supervision–Fourier Branch

**Figure 5:**
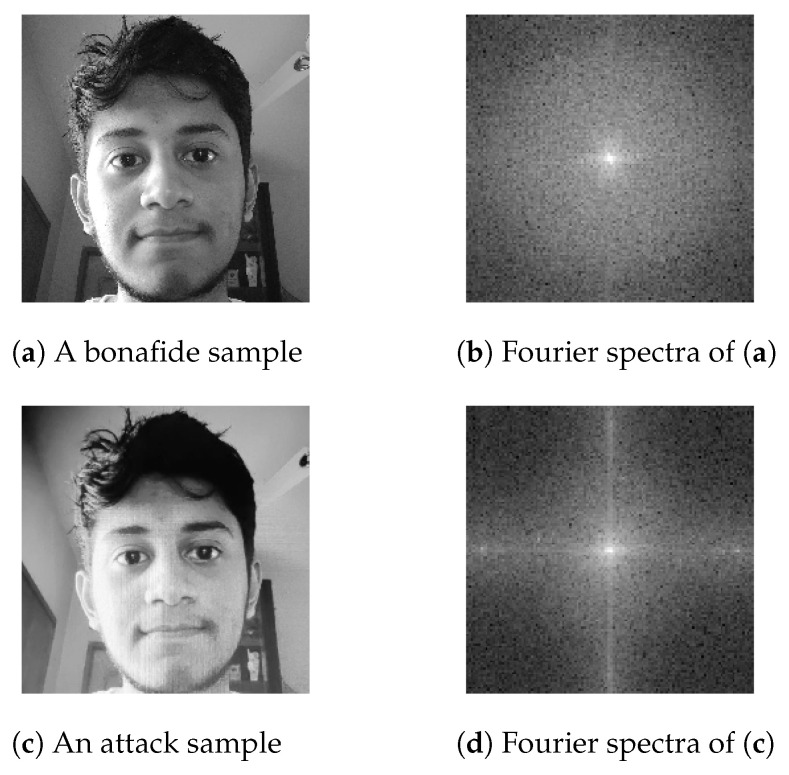
Visualizations of the 2D Fourier spectra for the attack and bonafide classes. Figure (**a**) represents a bonafide sample and (**b**) illustrates its Fourier spectra; Figure (**c**) portrays an attack sample captured through a video replay from a monitor screen, and (**d**) represents its Fourier spectra.

We further hypothesized that, particularly in the problem of FAS, unlike a bonafide sample, the 2D Fourier spectra of an attack sample would incorporate a lower number of high-frequency components, as shown in Figure 5. The paper proposed by [23] developed on the hypothesis that the number of high-frequency components of an attack sample must be very small. This is particularly true because for the sensor, when recording subjects in motion, the poses and expressions by those subjects become invariant or smoothened after being captured. Consequently, we leveraged the properties of 2D Fourier spectra by adding an auxiliary branch in our baseline BiFPN-based spoof detection model (Bi-FAS-S). Following the claims of [23], we further assumed that leveraging Fourier spectra would essentially inherit texture-based information from the input sample, which is crucial to the FAS task.

(12) $$F\left(x,y\right)=\sum_{i=0}^{N-1}\sum_{j=0}^{M-1}f\left(i,j\right)\;e^{-\iota (\omega_{x}i\;+\;\omega_{y}j)}$$

(13) $$\omega_{x}i=\frac{2\pi x}{N};\;\omega_{y}i=\frac{2\pi y}{M}$$

Firstly, we used the discrete Fourier transform (DFT) defined in Equation (12) to compute a sampled Fourier transform of the 2D input image. Although sampled, the frequency components embodied the bare minimum of components to distinguish among a variety of images. In Equation (12), f(i,j) represents the image in the spatial domain, and the basis functions $\omega_{x}i$ and $\omega_{y}j$ are defined in Equation (13). As the Fourier coefficients were relatively large, we used the logarithmic operator defined in Equation (14) for the zero-frequency component to shift towards the center of the spectrum.

(14) $$\hat{F}\left(x,y\right)=log\left(abs\left(\varphi \left(F\left(x,y\right)\right)\right)+1\right):\forall x\forall y$$

The $\hat{F}\left(x,y\right)$ represents the center-shifted Fourier coefficients of the image depicted on the frequency domain, where φ is the shift operator. From Figure 5, we can compare the spectrum distribution of a spoofed sample and a bonafide sample to better understand their distinctions.

Upon close inspection of Figure 5, we can observe a clear distinction between the Fourier spectra of the bonafide sample and the attack sample. Figure 5b is visually brighter than Figure 5d, which aligns with our hypotheses that the Fourier spectra of a bonafide sample should comprise a higher number of high-frequency components than the Fourier spectra of an attack sample, which should lead to a higher standard deviation in the bonafide class.

We modified the Bi-FAS model devised in the previous section to add another branch, with the objective of training the model with semantic information derived from the frequency domain of the image alongside textural cues generated from the BiFPN pyramids. We employed a generator based on a convolutional neural network Λ, which reconstructed the Fourier spectra of the input sample *S* and performed batch normalization [46] (BN), as shown in Equation (15), and further optimized the network in a self-supervised approach by using the loss functions defined in Equations (16) and (17).

(15) $$G_{i}=ReLU\left(BN\left(\Lambda \left(S\right)\right)\right):\forall i\;\left(i\in \left\{3,4,5\right\}\right)$$

**Figure 6:**
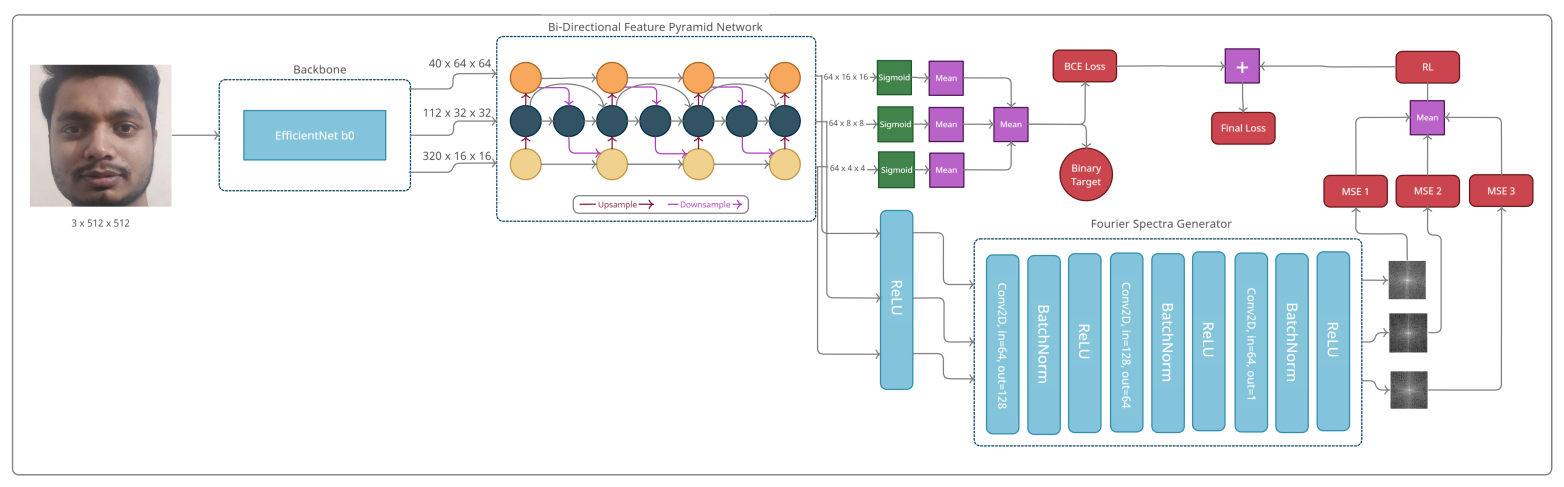
Extension of our baseline BiFPN architecture with a convolutional generator and reconstruction loss terms for the BiFPN-based spoof detection model (Bi-FAS-S).

With regards to the architecture presented in Figure 6, we generated the 2D Fourier spectra of the 512 × 512 dimensional gray-scaled input sample as our ground truth. Thus, we used the convolutional generator in Figure 6 for the output pyramids, $P_{3}$, $P_{4}$, and $P_{5}$, each reconstructed for the ground-truth Fourier spectra *S*, presented as $G_{3}$, $G_{4}$, and $G_{5}$, assuming that they would contain multi-scaled information with textural cues in the frequency domain, as previously demonstrated by [23]. During training, the goal of the generator was to provide texture-based information in the form of the Fourier spectra as an added cue for supervision. Due to this, the effectiveness of this branch was limited only during the training phase, and the generator was made inactive during inference.

(16) $$RL=\frac{1}{3*N}\left\{\sum_{i=1}^{N}{\left(S-G_{3}\right)}^{2}+{\left(S-G_{4}\right)}^{2}+{\left(S-G_{5}\right)}^{2}\right\}$$

(17) $$l=\{-\frac{1}{N}\sum_{i=1}^{N}t_{i}\;\cdot \;log\left(s_{i}\right)+\left(1-t_{i}\right)\;\cdot \;log\left(1-s_{i}\right)\}+RL$$

We used a reconstruction loss (RL), as defined in Equation (16), to optimize the reconstructions of the Fourier spectra generated from the three pyramids. The RL is a mean squared error loss function of the three generated Fourier spectra and uses the mean of the three terms on the binary cross-entropy loss defined in Equation (17).

Finally, we trained each of our models for two epochs, as the prior study showed that spoof detection models suffer due to over-parameterization, which eventually leads to overfitting [18], resulting in increased error rates and reducing the generalizability of the models; from our experiments, we also found that proceeding with further training resulted in deteriorated ACER scores.

## 5. Experiments and Results

In this section, we outline the experimental setup used to conduct our experiments; then, we describe the results of the two proposed methods on the OULU-NPU and Replay-Mobile datasets. In accordance with previous works [16,17,33], we present the results of the intra-dataset evaluation and subsequently compare the results of the inter-dataset testing. We compare our models primarily with the currently published pixel-wise architectures and also compare with other approaches based on popular algorithms.

### 5.1. Experimental Setup

First of all, we used the RetinaFace [44] face detection model to extract the face crop from the images. Due to the improved results, we extended the bounding boxes of RetinaFace to make the face crops square in shape. During training, we applied horizontal flip transformation randomly to 50% of the samples. We also applied color jitter randomly to augment the samples in the training set. We initialized our EfficientNet backbone feature extractor with the pre-trained ImageNet weights, and all other weights in the network were initialized using the Xavier Initialization [47]. For optimization, we employed the Adam optimizer, used a learning rate of $1\times e^{-4}$, and set the weight decay to $1\times e^{-5}$. We set a mini-batch size of 64 on eight Tesla K80 GPUs and selected the model based on the best ACER metric on the validation set. For both of our proposed architectures, we followed the same training, testing, and validation strategies as per the protocols specified in the dataset papers [6,14].

### 5.2. Intra-Dataset Testing

In this section, we present the results of our evaluation on the respective testing sets of the OULU-NPU and the Replay-Mobile datasets. We carefully followed the model training procedures of [6,14,16,17] for all the results that we present in this section and compare these results primarily with the pixel-wise supervised approaches [16,17]. Furthermore, during intra-dataset testing, for each protocol of the OULU-NPU dataset and the grandtest protocol of the Replay-Mobile dataset, we trained independent models for each of our two proposed architectures. Table 3 gives a comparison of the results of the two models on the OULU-NPU dataset. Other than Protocol I, on all other protocols, we found that the pyramid-based approach significantly outperformed the prior pixel-wise techniques. From Table 3, we can further observe that the addition of a self-supervised auxiliary branch that reconstructed the pyramid features for the original image in the frequency domain provided salient information and even outperformed the base model.

From Table 3, although we obtained an ACER of 0.49 on Protocol III, which is, by itself, extremely competitive, as the hardest protocol of the OULU-NPU dataset, we particularly took note of Protocol IV, on which we obtained a mean ACER of 2.92, which is the “lowest” in the currently available published research using pixel-wise supervision and 58% lower than our Bi-FAS approach.

Moreover, the ACER of our Bi-FAS-S model on Protocol IV is very similar to the ACER score of the NAS-FAS [51] model on the same testing set. However, the NAS-FAS model accomplished this task using a Neural Architecture Search, which tends to be computationally expensive, hence accumulating difficulty in deployment in low-powered devices.

From Table 4, we also find competitive results on the Replay-Mobile grandtest protocol, which are similar to the metrics achieved by other pixel-wise approaches, achieving an HTER of 0. Next, we used the Replay-Mobile grandtest protocol to perform the inter-dataset evaluations, as shown in the following section in Table 5.

### 5.3. Inter-Dataset Testing

In order to assess the generalizability of our models, we performed an inter-dataset evaluation over the combination of Protocol I of the OULU-NPU dataset with the grandtest protocol of the Replay-Mobile dataset. To elaborate, we conducted training on Protocol I of the OULU-NPU dataset and tested it on the grandtest protocol of the Replay-Mobile dataset, and vice-versa, as done in previous works [16,17,18,33]. For the OULU-NPU inter-dataset evaluation, we particularly chose Protocol I due to the size of the dataset and because this protocol has been used by most papers [16,17,33] for this evaluation task.

To this end, as seen in Table 5, we can see that our Bi-FAS and Bi-FAS-S models performed slightly better than the DeepPixBis and the A-DeepPixBis models when they were trained on Replay-Mobile and tested on OULU-NPU. However, when trained on Protocol I of OULU-NPU, we also found that the performance of the Bi-FAS model was inferior to those of the DeepPixBiS and A-DeepPixBis models, and the Bi-FAS-S model outperformed the DeepPixBis model. The Bi-FAS-S model performed better when trained on OULU-NPU (Protocol I) and tested on Replay-Mobile, mainly due to the presence of a wide variation of data present in the protocol, which further reinforces our claim of generalizability.

### 5.4. Result Analysis

Here, we provide an additional analysis of the results presented earlier in Table 3, Table 4 and Table 5. We first investigated cases where our Bi-FAS-S improved when compared with our baseline Bi-FAS model. We also analyzed some incorrect samples produced by the better-performing Bi-FAS-S model. Next, we discuss samples comprised of bonafide and attack samples and look into the differences in the pyramids and generated Fourier spectra of the Bi-FAS-S model, thus illuminating clear differences between the two classes.

In order to perform a qualitative analysis of the two Bi-FAS and Bi-FAS-S models, we took the logits of the three pyramids into account. For this analysis, we picked the largest pyramid $P_{3}$ from the two models and detected the samples on which the Bi-FAS-S operated correctly, but the Bi-FAS model was incorrect.

**Figure 7:**
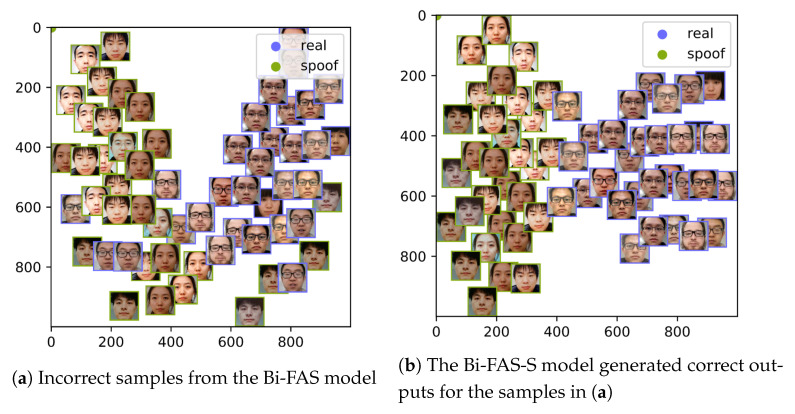
Visualizations of the *P*_3_ outputs of the incorrect samples from the Bi-FAS model and indications that they were corrected by the Bi-FAS-S model on Protocol I of OULU-NPU [14].

To perform the analysis shown in Figure 7, we started by determining all of the incorrect samples generated by the Bi-FAS model on Protocol I of the OULU-NPU dataset. We passed these incorrect samples over to our better-performing Bi-FAS-S model and found that it generated correct outputs on all of the samples provided. We then used the t-SNE algorithm [52] to make lower-dimensional points of the feature in $P_{3}$ of these samples, and they are presented in Figure 7a,b. Essentially, we used the high-dimensional feature of $P_{3}$, reduced it to a two-dimensional point [52], and plotted this on a two-dimensional plane, as shown in Figure 7, where the two axes represent the y and x coordinates of the low-dimensional $P_{3}$ pyramid. In Figure 7a, we can observe an intersection of the samples; however, in Figure 7b, we can notice a clear decision boundary between the two classes, which effectively leads to the premise that the Bi-FAS-S model performs better than its preceding form.

**Figure 8:**
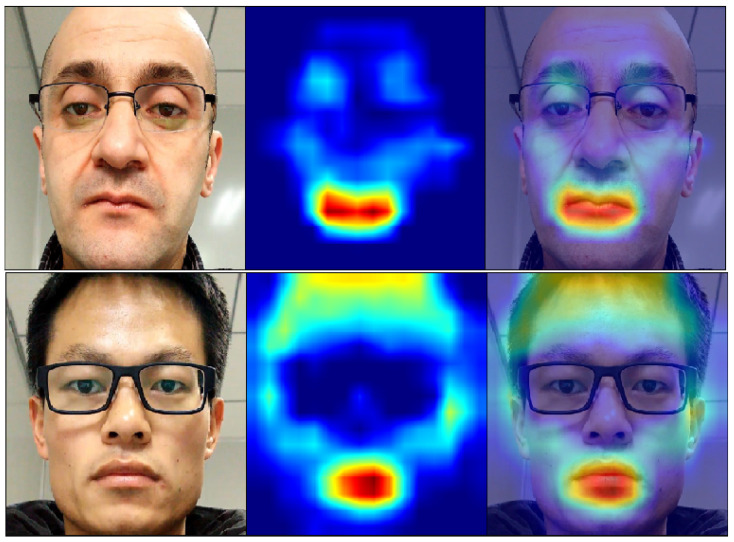
GRAD-CAM [53] visualizations of the last pyramid layer of the Bi-FAS-S model on the incorrect bonafide samples.

Protocol IV of the OULU-NPU dataset is by far the most challenging testing set among all of the experiments conducted in this paper. For this, we believe that it is appropriate to provide an analysis based on this partition. From Table 3, we can observe that the Bi-FAS-S model has a higher BPCER score than APCER, meaning that the model fails to classify bonafide samples more. The pattern shown in Figure 8 could be deduced from multiple incorrect bonafide samples when we leveraged GRAD-CAM [53] to visualize and examine the activations on the last convolutional layer of the $P_{3}$ pyramid. From Figure 8, we can see that the model had a higher activation region around the mouth, which points towards the claim that these regions were subject to the high BPCER score.

We believe that these specific cases could be resolved by employing a problem-specific augmentation methodology. However, in order to keep the experiments consistent, we opted not to include any additional image augmentations, as this could affect the consistency as well as the generalizability of the models.

Next, we inspected the patterns produced by the Bi-FAS-S model when tested on Protocol IV of the OULU-NPU dataset. We picked two samples from the bonafide and attack classes to first generate three heatmaps using the three pyramids, as well as to show the Fourier spectra generated using the convolutional Fourier spectrum generator, as shown in Figure 6.

**Figure 9:**
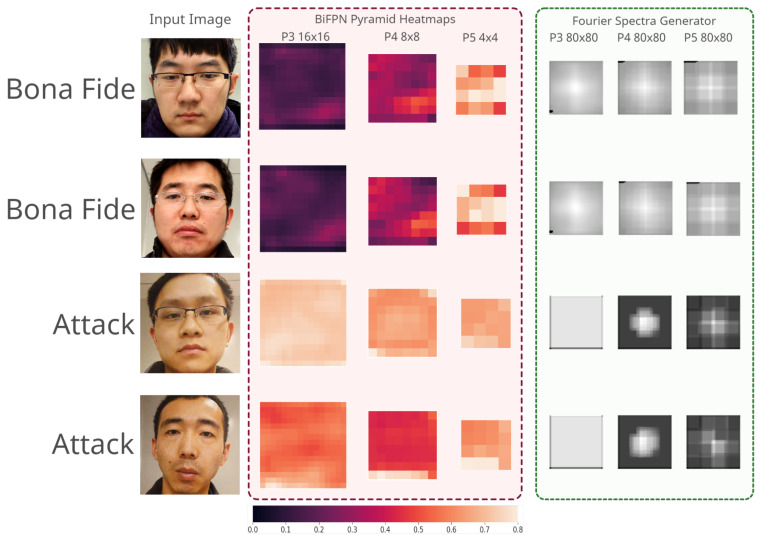
Heatmaps and Fourier spectra generated using three pyramids of the Bi-FAS-S model on four samples from the OULU-NPU [14] dataset.

Observing Figure 9, we generated heatmaps from the three pyramids of the Bi-FAS-S model, where each pixel refers to a probability score, as we used the pixel-wise approach with this model. A darker color on the heatmaps refers to the degree of the realness of the sample, whereas a lighter color refers to an inclination towards the attack sample. It can be clearly noticed that while $P_{3}$ and $P_{4}$ function ideally for both of the classes, $P_{5}$ was a bit unstable, as it consisted of multiple pixels that seemed to lean towards the spoof class.

In the right module in Figure 9, we present three 80 × 80 Fourier spectra generated by the convolutional generator. We found a clear distinction between the bonafide samples and the attack samples. However, we found that in the case of the bonafide samples, the model generated visible spectra, but generated solid or “almost” solid spectra, which potentially corroborates our hypothesis that the Fourier spectrum for a bonafide sample should contain a higher number of high-frequency components and higher standard deviations, where, in contrast, an attack sample would hold the opposite.

## 6. Discussion

In this section, we look into the positives as well as the negatives of our proposed architectures. Next, we compare the architectural differences of our Bi-FAS and the Bi-FAS-S models with the popular pixel-wise models for FAS. We finally discuss the significance of using a face detector and further elaborate on some challenges posed by the datasets we used and how they affected the inferences of our FAS models.

Firstly, we describe the differences in the architecture of our proposed Bi-FAS and Bi-FAS-S models with the popular pixel-wise models, namely, A-DeepPix and DeepPix [16,17]. Both the DeepPix and A-DeepPix models use the DenseNet [34] backbone to retrieve a feature map of size 14 × 14 for pixel-wise supervision. In contrast, in our approach, we use the EfficientNet [21] backbone, as it integrates readily with the BiFPN module. The main difference between the DeepPix and the A-DeepPix models is the introduction of an angular constraint on the conventional binary cross-entropy loss function, which is used in both the pixel-wise supervision branch as well as the classification branch. However, in this paper, we used the binary cross-entropy loss function, similarly to the DeepPix paper, but we applied it over the pixels of the three pyramids rather than using a 14 × 14 feature map. In addition to this, we also added an auxiliary supervision branch that optimizes the model based on its capability of reconstructing the Fourier features of the input sample. This added modality of supervision was also not investigated in the compared pixel-wise papers.

One of the significant positives that we found through our proposed models was the achievement of extremely competitive scores on Protocols III and IV of the OULU-NPU [14] dataset. This is important because Protocol IV is the most difficult testing partition of this dataset. Next, to demonstrate further generalizability, we achieved outstanding scores while conducting inter-dataset testing on the grandtest protocol of the Replay-Mobile dataset [6] and one of our self-acquired datasets (Appendix A). For these inter-dataset tests, the Bi-FAS and the Bi-FAS-S architectures were trained on Protocol I of the OULU-NPU dataset, as done by [16,17]. Additionally, in the Bi-FAS-S model, we used the features of the Fourier spectra of the image as an added form of supervision during training. While using depth-based features for additional supervision [33] may seem to be the preferable choice, generating Fourier features, as done in this paper, is less computationally expensive than generating depth features. This would essentially result in faster computation during the training phase.

In this paper, we used the RetinaFace [44] face detector for face localization and cropping. It can be argued that leveraging this component would increase the computational complexity of the pipeline, whereas an end-to-end approach could have led to further optimization and possibly an improvement in performance. However, an end-to-end approach would require a large quantity of data for the bonafide and attack classes, with a significant variation in the scenarios and background conditions; the amount of data publicly available for FAS is nowhere near what would be needed. On the other hand, using a pre-trained detector to locate faces means that the need for variability in scenarios is eliminated as background information is discarded. The use of a pre-trained face detector, however, makes the task simpler to handle, but carries all the associated issues. Next, we show examples that underline these issues more clearly to show how the usage of our face detector affects our FAS pipeline.

### Dataset Issues

As previously noted, we used a face detector to extract the faces from a full-framed image. Due to this dependency, one such shortcoming of this model arises, which essentially leads to the conclusion that our proposed models would operate optimally when using a cropped frontal face.

**Figure 10:**
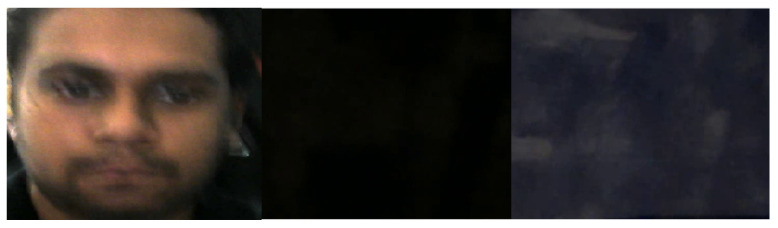
Samples indicating cases where the face detector failed to extract the face crops.

Considering the samples shown in Figure 10, we found that on multiple occasions, the face detection pipeline would fail to localize the face due to the samples having either motion blurriness or merely not containing a visible face. If we rejected the samples where RetinaFace fails to find a face from the frame, it would be sufficient to make a robust and potentially deployable FAS model.

## 7. Conclusions

In this paper, we looked into the problem of face anti-spoofing, which is commonly used with face recognition technologies. We employed a bi-directional feature pyramidal network to extract features of multiple scales. We initially found that the multi-scaled features from the BiFPN potentially consisted of texture-based cues, one of the dominant attributes for a spoofed image. Next, we hypothesized that, upon transforming an image into the frequency domain, the number of high-frequency components for a bonafide image would be significantly higher than a that for a spoofed image. Following these two ideas, coupled with the pixel-wise approaches from the DeepPixBis paper, we proposed two architectures.

In the first model, we computed the features from the EfficientNet backbone and further used it to extract multi-scaled features from the BiFPN. Despite using all five pyramids from the BiFPN, in our experiments, we abandoned the two high-level pyramids, as they did not contribute to improving the results. A sigmoid operation was performed over all of the pixels of the three pyramids, after which we computed the mean of the probability scores, which determined the final probability of the sample being a bonafide image.

For the second approach, using the first model as a baseline, we added a self-supervised auxiliary branch that used multiple convolutional operations and reconstructed the outputs of the three pyramids into the original image in its frequency domain. According to the evaluation strategies of prior works, our two proposed approaches showed competitive results on the OULU-NPU and Replay-Mobile datasets. We particularly found that our second approach obtained an ACER of 2.92% on Protocol IV of the OULU-NPU dataset, which is currently the highest score among all of the published works. We also performed inter-dataset testing on the OULU-NPU and Replay-Mobile datasets to confirm that with the inclusion of a wide variety of data, our model would generalize well on an unseen test set with various sensors.

In the future, we would like to explore our baseline approach further. We plan to experiment with angular-based constraints, enforcing the performance of multiple computations on the angular space according to the convention set by A-DeepPixBis. We would also like to explore methods where we leverage depth-based features, which, as in the CDCN paper, can be used for an additional form of supervision. We believe that these ideas would be helpful in contributing towards the problem of face anti-spoofing and would help to build solutions that would make FAS systems more functional and robust. 

## Figures and Tables

**Table 1:** OULU-NPU datasets [14,17].

Protocol	Subset	Session	Phones	User	# Attacks Created Using	# Real Videos	# Attack Videos	# All Videos
I	Train	Session 1,2	6 Phones	1–20	Printer 1,2; Display 1,2	240	960	1200
	Dev	Session 1,2	6 Phones	21–35	Printer 1,2; Display 1,2	180	720	900
	Test	Session 3	6 Phones	36–55	Printer 1,2; Display 1,2	120	480	600
II	Train	Session 1,2.3	6 Phones	1–20	Printer 1; Display 1	360	720	1080
	Dev	Session 1,2.3	6 Phones	21–35	Printer 1; Display 1	270	540	810
	Test	Session 1,2.3	6 Phones	36–55	Printer 2; Display 2	360	720	1080
III	Train	Session 1,2.3	5 Phones	1–20	Printer 1,2; Display 1,2	300	1200	1500
	Dev	Session 1,2.3	5 Phones	21–35	Printer 1,2; Display 1,2	225	900	1125
	Test	Session 1,2.3	1 Phones	36–55	Printer 1,2; Display 1,2	60	240	300
IV	Train	Session 1,2	5 Phones	1–20	Printer 1; Display 1	200	400	600
	Dev	Session 1,2	5 Phones	21–35	Printer 1; Display 1	150	300	450
	Test	Session 3	1 Phones	36–55	Printer 2; Display 2	20	40	60

**Table 2:** Resolutions of the five convolutional feature pyramids.

Pyramid	Resolution
$P_{1}$	64 × 64 × 64
$P_{2}$	64 × 32 × 32
$P_{3}$	64 × 16 × 16
$P_{4}$	64 × 8 × 8
$P_{5}$	64 × 4 × 4

**Table 3:** Metrics of our proposed models compared with other algorithms on OULU-NPU [14] for intra-dataset testing.

Protocol	Model	APCER (%)	BPCER (%)	ACER (%)
	CPqD [48]	2.9	10.8	6.9
	GRADIANT [48]	1.3	12.5	6.9
	FAS-BAS [49]	1.6	1.6	1.6
	IQM-SVM [50]	19.17	30.83	25.0
1	LBP-SVM [16]	12.92	51.67	32.29
	DeepPixBiS [16]	**0.83**	**0.0**	**0.42**
	A-DeepPixBis [17]	1.19	0.31	0.75
	**Bi-FAS (ours)**	2.92	3.33	3.12
	**Bi-FAS-S (ours)**	3.13	0.83	1.97
	MixedFASNet [48]	9.7	2.5	6.1
	FAS-BAS [49]	2.7	2.7	2.7
	GRADIANT [48]	3.1	1.9	2.5
	IQM-SVM [50]	12.5	16.94	14.72
2	LBP-SVM [16]	30	20.28	25.14
	DeepPixBiS [16]	11.39	0.56	5.97
	A-DeepPixBis [17]	4.35	1.29	2.82
	**Bi-FAS (ours)**	2.36	**1.11**	1.73
	**Bi-FAS-S (ours)**	**1.67**	**1.11**	**1.39**
	MixedFASNet [48]	5.3±6.7	7.8±5.5	6.5±4.6
	GRADIANT [48]	2.6±3.9	5.0±5.3	3.8±2.4
	FAS-BAS [49]	2.7±1.3	3.1±1.7	2.9±1.5
	IQM-SVM [50]	21.94±9.99	21.95±16.79	21.95±8.09
3	LBP-SVM [16]	28.5±23.05	23.33±17.98	25.92±11.25
	DeepPixBiS [16]	11.67±19.57	10.56±14.06	11.11±9.4
	A-DeepPixBis [17]	2.78±3.47	11.16±16.45	6.97±7.57
	**Bi-FAS (ours)**	2.92±2.30	1.11±1.72	2.01±1.70
	**Bi-FAS-S (ours)**	**0.69 ± 0.68**	**0.28 ± 0.68**	**0.49 ± 0.63**
	MassyHNU [48]	35.8±35.3	8.3±4.1	22.1±17.6
	GRADIANT [48]	5.0±4.5	15.0±7.1	10.0±5.0
	FAS-BAS [49]	9.3±5.6	10.4±6.0	9.5±6.0
	IQM-SVM [50]	34.17±25.89	39.17±23.35	36.67±12.13
4	LBP-SVM [16]	41.67±27.03	55.0±21.21	48.33±6.07
	DeepPixBiS [16]	36.67±29.67	13.33±16.75	25.0±12.
	A-DeepPixBis [17]	3.86 ± 4.04	6.56±7.88	5.22±2.96
	**Bi-FAS (ours)**	8.75±8.12	5.00±6.32	6.88±2.82
	**Bi-FAS-S (ours)**	**2.50 ± 3.16**	**3.33 ± 4.08**	**2.92 ± 3.41**

**Table 4:** Performance comparison of our proposed approach with other popular methodologies on the Replay-Mobile grandtest protocol [6].

Model	EER (%)	HTER (%)
IQM-SVM [50]	1.2	3.9
LBP-SVM [16]	6.2	12.1
**DeepPixBiS [16]**	**0.0**	**0.0**
**A-DeepPixBis(binary output) [17]**	**0.0**	**0.0**
**A-DeepPixBis(feature map) [17]**	**0.0**	**0.0**
**Bi-FAS (ours)**	**0.0**	**0.0**
**Bi-FAS-S (ours)**	**0.0**	**0.0**

**Table 5:** Inter-dataset comparison of our proposed models on Protocol I of the OULU-NPU dataset and the Replay-Mobile grandtest protocol represented using half-total error rate (HTER) values in percentages (%).

	Trained on OULU		Trained on Replay-Mobile	
**Model**	**Tested on OULU**	**Tested on Replay-Mobile**	**Tested on OULU**	**Tested on Replay-Mobile**
IQM-SVM [50]	24.6	31.6	**3.9**	42.3
LBP-SVM [16]	32.2	35.0	12.1	43.6
DeepPixBiS [16]	0.4	12.4	22.7	0.0
**A-DeepPixBis [17]**	0.7	**9.35**	25.57	0.0
**Bi-FAS (ours)**	3.12	18.91	18.33	0.0
**Bi-FAS-S (ours)**	1.97	**11.97**	21.24	0.0

**Figure A1:**
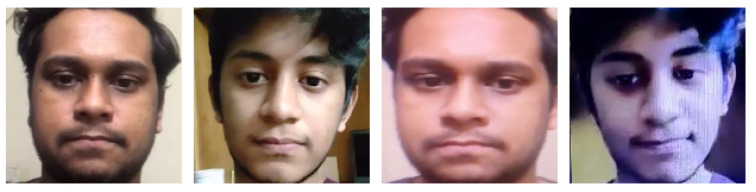
A few frames extracted from the videos of our self-acquired dataset.

## Data Availability

The data presented in the Appendix A are available on request from the corresponding author.

## Appendix A. Evaluation on a Self-Acquired Dataset

In this section, we provide information on the experiments that we conducted on our self-acquired dataset to further demonstrate the generalizability of our proposed architectures. Due to physical constraints, we assembled this dataset with three subjects. All of the videos captured in this dataset were captured with the iPhone 7, OnePlus 7, and OnePlus Nord camera sensors. All of the images were captured within an indoor setting and consist of only a testing partition. The attack samples were comprised of only replay attacks from a BenQ monitor and a Macbook Air display. Each of the bonafide and attack videos were recorded for 10 s in length with in an attempt to integrate multiple orientations of faces as well as to add natural artifacts, such as motion blur and glare from indoor lights. In total, the dataset consisted of around six videos and extracted 1003 bonafide frames and 1469 attack frames, which were further used to evaluate our proposed models.

Figure A1 depicts a sample of images taken from our self-acquired dataset. In the figure, the two leftmost samples of subject 1 and 2, respectively, denote the bonafide class, and the last two images represent the attack/spoof class. We tested our pre-existing models on this dataset and report our results in Table A1. For this evaluation, similarly to Table 4, we leveraged the two models trained on Protocol I of the OULU-NPU dataset. We would like to reiterate that we selected this protocol for our trained models because this partition consists of the maximum number of training samples of all of the protocols, and other popular papers [16,17,33] used the same protocol for the trained models in such evaluations.

**Table A1:** Performance of our proposed architecture on our self-acquired dataset and trained on Protocol I of the OULU-NPU dataset.

Model	APCER (%)	BPCER (%)	ACER (%)
Bi-FAS	16.26	13.75	15.01
Bi-FAS-S	14.01	14.29	14.17

Table A1, gives a quantitative overview of the performance of the two architectures proposed in this paper. Our Bi-FAS-S model achieved an ACER, APCER, and BPCER of 14% on this dataset, which, by itself, indicates that for both the bonafide and attack classes, the model correctly predicted 86% of the cases. On the other hand, our plain Bi-FAS model achieved an ACER of 15.01%, from which it can be concluded that it performed correctly on 85% of the samples. Both the ACER scores of the two models—albeit extremely close—consisted of a large variation when compared with the metrics of the OULU-NPU evaluations in Table 3.

From this analysis, we can arrive at several conclusions. Firstly, we believe that achieving an ACER of 15% and 14% on a testing set with a distribution that is entirely unknown for the model is a reasonable achievement. Secondly, although we achieved satisfactory metrics on our self-acquired dataset, it is not guaranteed that the performance of our model on completely unknown testing sets would be proportional to the performance achieved on a benchmark dataset.

The self-acquired dataset that we used for evaluation will be available on request for conducting further research and performance comparisons.
